# Defect States in the Electronic Structure of Ga_2_O_3_ Transparent Conductive Oxide Surfaces

**DOI:** 10.1021/acs.jpcc.6c02301

**Published:** 2026-06-23

**Authors:** Elizaveta Pyatenko, Constantin Wansorra, Ralph Steininger, John Vinson, Wolfram Witte, Dimitrios Hariskos, Michael Powalla, Clemens Heske, Lothar Weinhardt, Dirk Hauschild

**Affiliations:** † Institute for Photon Science and Synchrotron Radiation (IPS), Karlsruhe Institute of Technology (KIT), Kaiserstraße 12, 76131 Karlsruhe, Germany; ‡ Department of Chemistry and Biochemistry, University of Nevada, Las Vegas (UNLV), 4505 Maryland Parkway, Las Vegas, Nevada 89154-4003, United States; § Material Measurement Laboratory, 96988 National Institute of Standards and Technology (NIST), Gaithersburg, Maryland 20899, United States; ∥ Zentrum für Sonnenenergie- und Wasserstoff-Forschung Baden-Württemberg (ZSW), Meitnerstraße 1, 70563 Stuttgart, Germany; ⊥ Institute for Chemical Technology and Polymer Chemistry (ITCP), Karlsruhe Institute of Technology (KIT), Kaiserstraße 12, 76131 Karlsruhe, Germany

## Abstract

Defect states are crucial in many electronic devices. Oxygen vacancies, for example, are common in transparent conductive oxides and can both be beneficial (e.g., as a dopant to enhance conductivity) as well as detrimental (e.g., leading to reduced device performance by recombination at interfaces). Using soft and hard X-ray photoelectron spectroscopy (PES and HAXPES) with excitation photon energies ranging from 0.1 to 6.3 keV, we study the electronic surface structure of differently processed Ga_2_O_3_ samples in a depth-resolved fashion. Specifically, we investigate a Ga_2_O_3_ thin film, as used in Cu­(In,Ga)­Se_2_-based thin-film solar cells, as well as a β-Ga_2_O_3_ single crystal before and after a defect-inducing Ar^+^-ion treatment. Spectra calculations based on density functional theory (DFT) are used to explain the PES and HAXPES valence band signatures as a function of the excitation photon energy. The β-Ga_2_O_3_ single crystal spectra can be well described by the DFT calculations. In contrast, the Ga_2_O_3_ thin film and Ar^+^-ion treated β-Ga_2_O_3_ show significant spectral broadening of the valence band features and additional spectral intensity close to the valence band maximum, which we assign to defect states. This additional intensity varies as a function of probing depth, suggesting that these defects are mostly localized at the surface. We also discuss the importance of DFT-based spectra calculations to verify the proper determination of valence band maxima using a linear extrapolation.

## Graphical abstract

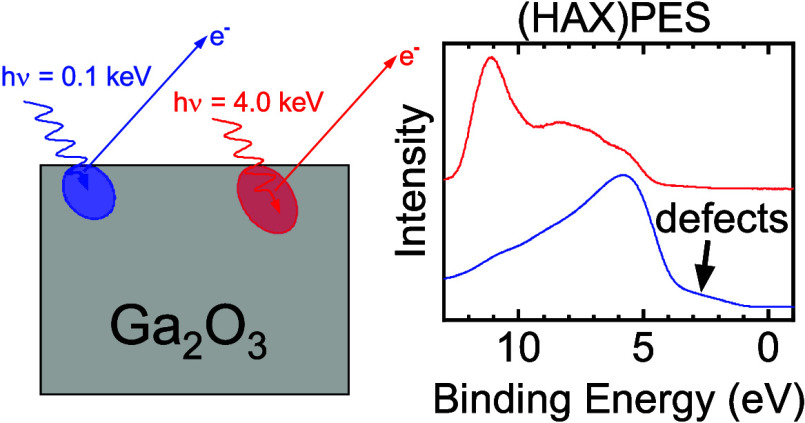

## 1 Introduction

Transparent conductive oxides (TCOs) are used in a multitude of optoelectronic applications. Defect states play a crucial role in these applications and critically impact the performance of the corresponding devices. For instance, the n-type conductivity of TCOs is often attributed to a high density of defect states, e.g., oxygen vacancies, cation interstitials, and hydrogen-doping, which introduce shallow donor-like states.^1–3^ However, unwanted defects, e.g., at interfaces, can also lead to an increased charge-carrier recombination, which, in turn, diminishes the device performance.^4^ In an earlier study on zinc magnesium oxide, we identified how defect-derived states appear as additional spectral intensity close to the valence and conduction band edges (“tail states”) in photoelectron spectroscopy data.^5^

Gallium oxide (with the most stable crystalline phase β-Ga_2_O_3_) is a TCO with a bandgap >4.5 eV^6^ and is used in power electronics, solar-blind UV detectors, and gas-sensing devices.^7–11^ Moreover, radio frequency (RF) magnetron sputter-deposited gallium oxide can be used as an electron transport and buffer layer in Cu­(In,Ga)­Se_2_ (CIGSe) thin-film solar cells.^12,13^ Ga_2_O_3_ shows a high intrinsic n-type conductivity, the origin of which is still under discussion.^14^ Several groups relate the conductivity to oxygen vacancies,^15,16^ but others found oxygen vacancies to be deep donors and concluded that the reason for the high conductivity is due to unintentional impurity doping with hydrogen.^3,17–19^

To improve the efficiency of TCO-containing devices, it is important to obtain a detailed understanding of their electronic structure, in particular the defect states and their impact on the (surface and interface) band edges. Here, we present a detailed study of the surface and near-surface bulk electronic structure of a “real-world” Ga_2_O_3_ film, i.e., used as a buffer layer in thin-film solar cells, and compare it with both a cleaved and an Ar^+^-ion treated β-Ga_2_O_3_ single crystal (SC) surface. We have performed soft and hard X-ray photoelectron spectroscopy (PES and HAXPES, respectively) using excitation photon energies ranging from 0.1 to 6.3 keV. This wide range of excitation photon energies makes it possible to study the valence band in a depth-resolved fashion, varying the electron inelastic mean free path (IMFP) of the photoelectrons (responsible for the depth sensitivity in photoemission) from ≈0.5 nm to ≈8 nm.^20,21^

## 2 Methods

A 100 nm Ga_2_O_3_ thin-film was deposited onto a CIGSe solar-cell absorber with RF magnetron sputtering at the Zentrum für Sonnenenergie- und Wasserstoff-Forschung Baden-Württemberg (ZSW) (this sample will be referred to as “Ga_2_O_3_/CIGSe”). More details on the sample preparation are available in refs 12,13 , and a detailed study of the chemical structure of the Ga_2_O_3_/CIGSe interface can be found in ref 22. Two β-Ga_2_O_3_ single crystals (Sn-doped at a concentration of 3.6 × 10^18^ cm^–3^; 10 mm × 15 mm × 0.5 mm; (010) orientation) were obtained from Novel Crystal Technology, Inc., Tamura Corporation.^23^ One of these single crystals was also used for a Ga_2_O_3_ resonant inelastic (soft) X-ray scattering (RIXS) study.^24^ The β-Ga_2_O_3_ SCs were cleaved in ultra-high vacuum immediately before the (HAX)­PES measurements, ensuring a clean surface. In addition, to induce surface defects, the second β-Ga_2_O_3_ crystal was treated with 50 eV Ar^+^-ions for 1 h at a current density of *j* ≈ 500 nA/cm^2^ using a FOCUS FDG 150 ion source in the Materials for Energy (MFE) laboratory at Karlsruhe Institute of Technology (KIT). Both the Ar^+^-treated β-Ga_2_O_3_ and the Ga_2_O_3_/CIGSe samples were transferred in an Ar-filled sample container without any air exposure from the MFE lab to the X-SPEC beamline at the KIT Light Source.^25^ The PES measurements (photon energies of up to 1.0 keV) were performed using X-SPEC’s focusing variable-line-space plane-grating monochromator (FVLS-PGM), while the HAXPES measurements were performed using the Si(111), Si(311), and Si(333) reflections of the double crystal monochromator (DCM) for 2.1, 4.0, and 6.3 keV, respectively. The binding energy scale was calibrated using the Au 4f_7/2_ peak and the Fermi edge^26^ of a sputter-cleaned Au foil. The experimental resolution was determined with the Fermi edge and varies between 100 and 300 meV for excitation photon energies between 0.1 and 6.3 keV, respectively.

Photoelectron spectra were simulated based on the projected density of state (PDOS) calculated with Wien2k, which solves the Kohn–Sham Hamiltonian based on the full-potential linearized augmented plane-wave + local orbital ((L)­APW + lo) method.^27^ The generalized gradient approximation (GGA), as parametrized by Perdew, Burke, and Ernzerhof (PBE), was used to approximate the exchange-correlation functional.^28^ The self-consistent field (SCF) cycle and the PDOS calculation were run with 1000 and 10,000 *k* points, respectively. The plane-wave cutoff parameter *R*_mt_·*K*_max_ (with *R*_mt_ being the smallest muffin-tin radius and *K*_max_ the maximum reciprocal space vector) was set to 7.0, and the SCF energy convergence threshold was set to 0.0001 Ry. The PES spectra were calculated with Wien2k’s PES module, weighting the PDOS with the excitation-energy-dependent atomic-orbital cross sections after taking the experimental geometry into account.^29–31^ The Ga 3d orbitals were treated as valence orbitals, together with Ga 4s, Ga 4p, O 2s, and O 2p. The crystal structure of β-Ga_2_O_3_ was obtained from the Materials Project Database.^32^

## 3 Results and Discussion

Figure 1 shows the valence band (HAX)­PES spectra of the Ga_2_O_3_/CIGSe thin-film sample and the β-Ga_2_O_3_ single crystals, after cleaving and after a 50 eV Ar^+^-ion treatment. The spectra are stacked as a function of the excitation photon energy (from 0.1 keV up to 6.3 keV). The spectral shape changes significantly as a function of excitation photon energy, as well as between the different samples. We first discuss the excitation energy dependence, followed by the spectral changes between the different samples.

**Figure 1:**
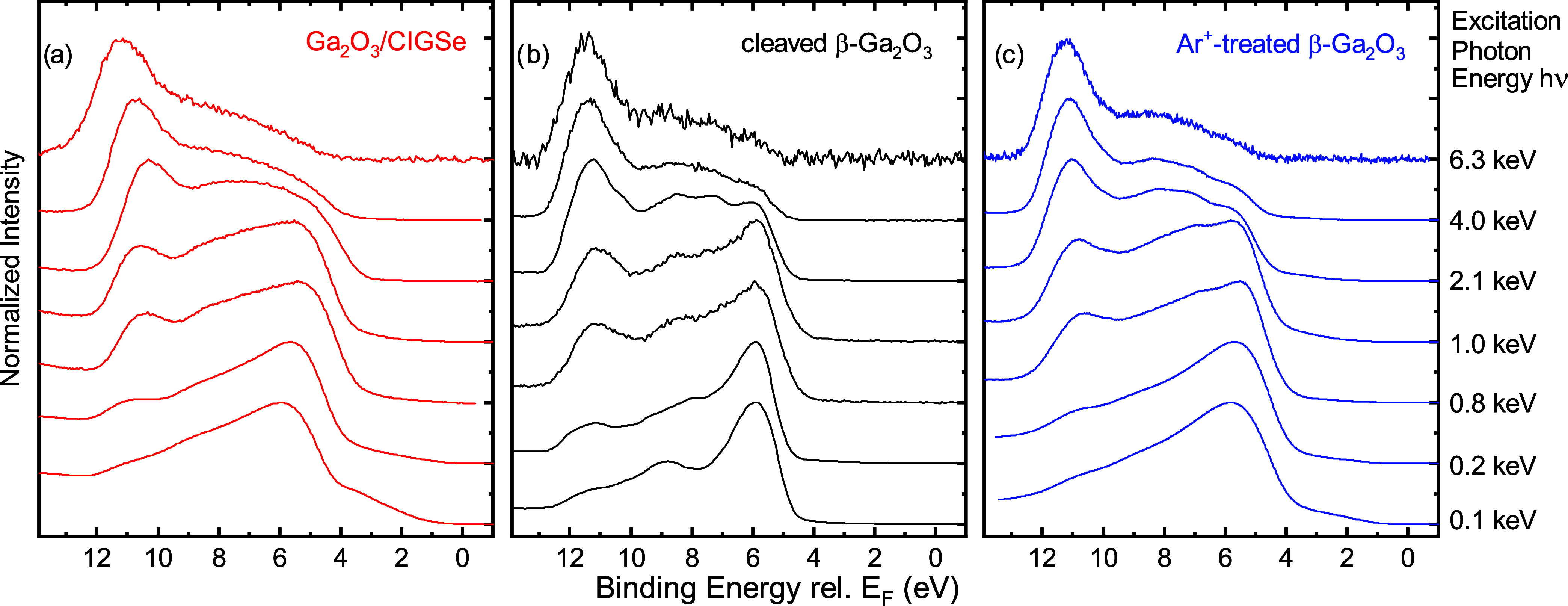
Photoemission valence band spectra relative to *E*_F_ as a function of excitation photon energy: (a) The Ga_2_O_3_/CIGSe thin-film sample (red), (b) the cleaved β-Ga_2_O_3_ single crystal (black), and (c) the β-Ga_2_O_3_ single crystal after Ar^+^-ion treatment (blue). Each spectrum is normalized to equal height of its maximum.

Two effects play a role for the excitation energy dependence of the spectra: first, measurements become increasingly surface sensitive with decreasing excitation energy, which lowers the kinetic energy of the emitted photoelectrons, thereby reducing their inelastic mean free path (IMFP, i.e., the 1/*e* attenuation length of the electrons). In Ga_2_O_3_, the IMFP is reduced from ≈8.0 nm at 6.3 keV to ≈0.5 nm at 0.1 keV.^20,21^ Due to this variation, depth-resolved information can be extracted from such a spectral series. Second, the photoionization cross sections vary strongly with excitation energy (and differently for different angular momenta of the valence states).

To illustrate this cross-section dependence, Figure 2 compares the experimental spectra of the cleaved β-Ga_2_O_3_ single crystal with DFT PES spectra calculations, separated into contributions from different valence states for *hv* = 0.2 and 2.1 keV. At 0.2 keV, the spectrum is dominated by contributions of states with O p and Ga d character, while contributions from states with Ga s, Ga p, and O s character are weak. In contrast, states with Ga s and Ga p character dominate the spectrum at 2.1 keV, with smaller contributions from Ga d, O p, and O s. These results are in line with earlier results in literature.^33,34^ To directly compare our calculations with the experimental data, the calculations were broadened with a Voigt profile using a Gaussian with full-width-at-half-maximum (FWHM) of 0.3 eV and a Lorentzian with a FWHM of 0.3 eV for 0.2 keV, while 0.5 and 0.3 eV were used for 2.1 keV, respectively. For best alignment with the experiment, both calculated energy scales were shifted toward higher binding energy by 4.75 eV and then stretched by a factor of 1.06. Overall, the calculated spectra in Figure 2 agree well with the experimental data, which demonstrates that—in the case of the cleaved single crystal—the spectral changes observed as a function of excitation energy can be primarily attributed to the changes in photoionization cross sections for different valence state characters (rather than any depth variation of the chemical composition and/or electronic structure).

**Figure 2:**
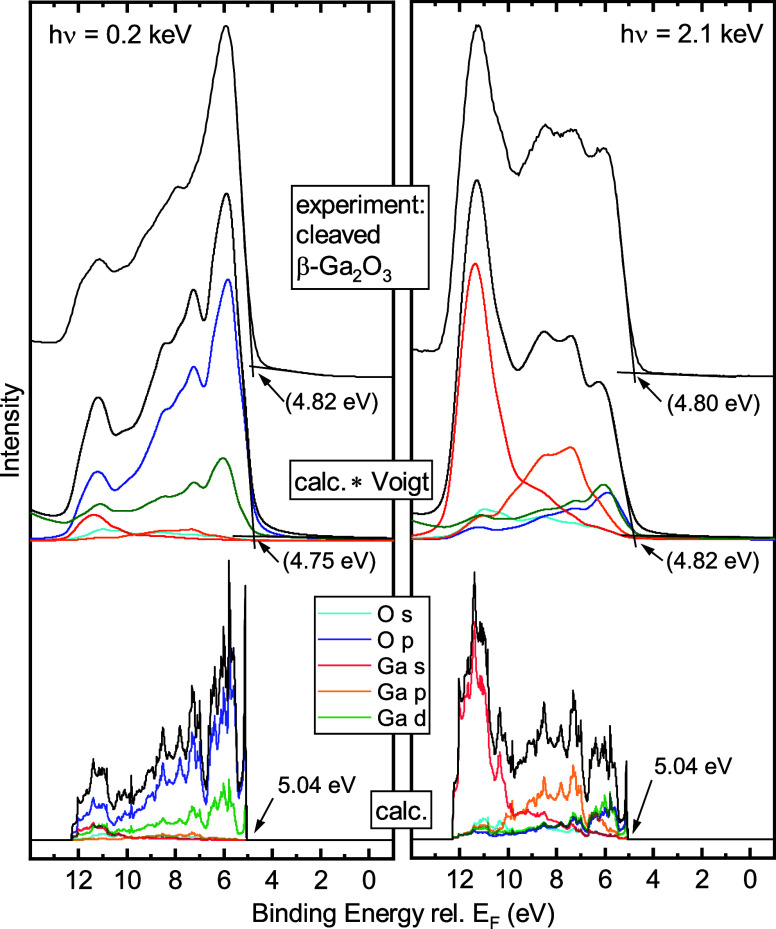
Valence band (HAX)­PES spectra of the cleaved β-Ga_2_O_3_ single crystal for *hv* = 0.2 keV (left) and 2.1 keV (right). From bottom to top: DFT-based spectra calculations, calculated spectra convoluted with a Voigt profile, and experimental spectra. For the calculated spectra, contributions of the different valence states are shown in color. Energy scales of both calculations were shifted toward higher binding energies by 4.75 eV and stretched by a factor of 1.06 for best alignment with the experiment. The valence band maximum (VBM) onsets from the shifted and stretched (but unbroadened) calculations, as well as approximate values determined by linear extrapolations of the leading edge for the broadened calculations and the experiments (values in parentheses) are listed next to the corresponding spectra.

As mentioned above, significant spectral differences are visible between the Ga_2_O_3_/CIGSe sample and the cleaved β-Ga_2_O_3_ single crystal in Figure 1a,b. First, all spectral features are broader and less defined in the Ga_2_O_3_/CIGSe sample (see discussion below). Second, additional intensity is observed between binding energies of 2 and 4 eV in the Ga_2_O_3_/CIGSe sample, especially for the low excitation photon energies (*hv* = 0.1 and 0.2 keV), which is neither found for the cleaved β-Ga_2_O_3_ single crystal nor in the calculations. As will be discussed in the following, we attribute this to a disordered and defect-rich Ga_2_O_3_/CIGSe sample surface. To test this assignment, the β-Ga_2_O_3_ single crystal was treated with 50 eV Ar^+^-ions (*j* ≈ 250 nA/cm^2^) for 1 h to disturb the surface structure and induce defects. While such low ion energies can be effectively used to remove unwanted surface adsorbates from applied material systems with only minimal additional changes to the surface (e.g., for sulfides and chalcopyrites),^35–38^ oxide surfaces are generally more sensitive to ions or X-rays,^39^ and metallic phases can be induced at the surface of a variety of compound semiconductors when employed for very long treatment times or higher ion energies.^35,37,40^ After the Ar^+^-ion treatment of the single crystal (Figure 1c), all features are significantly broadened in comparison to the untreated single crystal, and the overall spectral shape resembles that of the Ga_2_O_3_/CIGSe sample. For the low photon energy measurements, in particular for *hv* = 0.1 and 0.2 keV, a clearly visible spectral weight between 2 and 4 eV appears, which suggests that these Ar^+^-ion treatment-induced features are predominantly located at the ion-exposed surface (as would be expected).

As stated above, we attribute the Ar^+^-ion treatment-induced spectral changes to a more disordered surface structure, which leads to a broadening of all spectral features, similar to the Ga_2_O_3_/CIGSe sample. To analyze this, we broadened the spectra of the cleaved β-Ga_2_O_3_ single crystal excited at 0.1 and 2.1 keV, as shown in Figure 3. To achieve spectral similarity with the Ar^+^-ion treated β-Ga_2_O_3_ single crystal, the 0.1 and 2.1 keV data were convoluted with Gaussians with a fwhm of 1.8 eV and of 0.6 eV, respectively. The significantly larger FWHM for the surface-sensitive data implies that the Ar^+^-ion treatment has a stronger impact on the ion-exposed surface, as expected. The broadened spectra (green) for both the 0.1 and 2.1 keV data give a reasonable description of the spectra of the Ar^+^-treated β-Ga_2_O_3_ single crystal. However, the intensity in the region around 2 to 4 eV, which we attribute to defect states, is only visible for the Ar^+^-ion treated β-Ga_2_O_3_ single crystal and the Ga_2_O_3_/CIGSe sample (and not in the spectra of the cleaved β-Ga_2_O_3_ single crystal and hence also not in the broadened spectra). Overall, these states become more prominent for the more surface-sensitive measurements (see Figures 1 and 3), suggesting that they are localized near the surface due to acceptor-like defect states in Ga_2_O_3_.^19,41,42^ Interestingly, while these states are most prominent in the 0.1 keV data of the Ga_2_O_3_/CIGSe surface, their intensity drops more strongly than for the Ar^+^-ion treated β-Ga_2_O_3_ single crystal at higher excitation energies; in the Ga_2_O_3_/CIGSe 2.1 keV data, they are not visible at all. This indicates that, for the Ga_2_O_3_/CIGSe sample, the defect states are more localized at the surface, while they extend more into the surface-near bulk region in the case of the Ar^+^-ion treated β-Ga_2_O_3_ single crystal. We surmise that, in the Ga_2_O_3_/CIGSe sample, empirical process optimization minimizes the majority of defects in the bulk of the sample, which (still) enables high power-conversion efficiency in the photovoltaic device. One can speculate that the above-discussed defects also likely introduce additional states in the unoccupied electronic structure^43,44^ (not accessible by (HAX)­PES).

**Figure 3:**
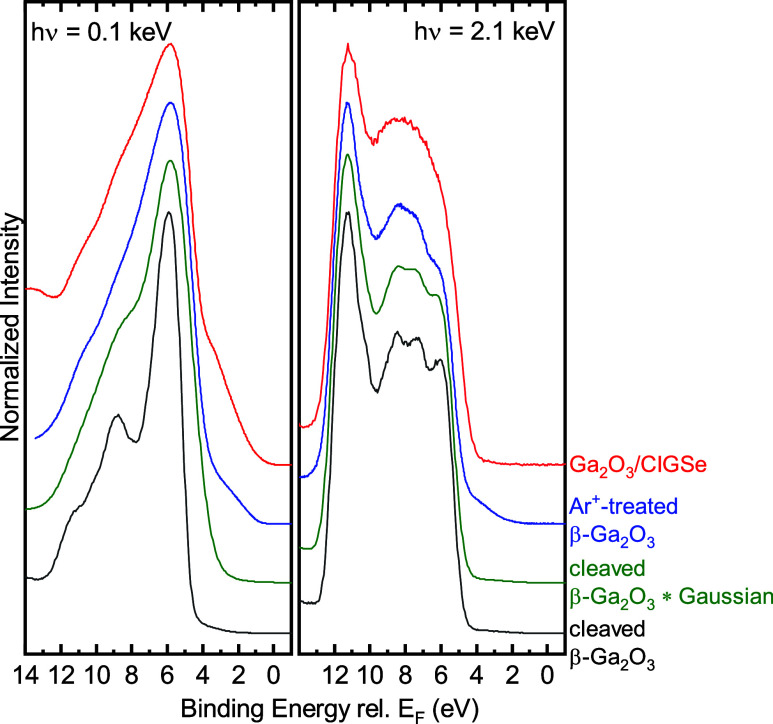
Valence band spectra of the cleaved β-Ga_2_O_3_ single crystal without (black) and with additional Voigt-broadening (green), the Ar^+^-ion treated β-Ga_2_O_3_ single crystal (blue), and the Ga_2_O_3_/CIGSe sample, for *hv* = 0.1 keV (left) and 2.1 keV (right). The Ga_2_O_3_/CIGSe sample spectrum was shifted by 0.10 eV toward lower binding energies for the best alignment with the other spectra.

Finally, we discuss the determination of the valence band maximum (VBM) for Ga_2_O_3_ samples. For this purpose, we directly compare the onset of the valence band in the experiments with those of the aligned unbroadened and Voigt-broadened calculations in Figure 2. To do this, the onsets in the experiment and the Voigt-broadened calculation were approximated by linear extrapolations of the leading edge; for the experimental data, this is intended to account for effects like inelastic scattering (e.g., phonon scattering), band dispersion, and final-state screening effects.^45^ The VBM onset directly read from the unbroadened calculations is found at 5.04 eV for both the 0.2 and 2.1 keV spectra, while the VBM determined by the linear extrapolations of the Voigt-broadened and β-Ga_2_O_3_ single-crystal spectra give 4.75 to 4.82 eV (see values in parentheses in Figure 2), the latter similar to other reported Ga_2_O_3_ VBM values.^33,46^ The difference of ≈0.2 to 0.3 eV likely arises from the shape of the PDOS of Ga_2_O_3_ that includes an intense and sharp peak close to the VBM, originating from O p and Ga d states. Broadening this sharp peak with a Voigt profile shifts the linear extrapolation significantly *above* the true VBM. For a pure Gaussian broadening and a linear extrapolation in the inflection point of the Gaussian, this shift would correspond to 2σ = 0.85 FWHM. As a consequence, the linear-extrapolation approach cannot yield the true valence-band onset in this case. In contrast, for materials with a PDOS that decreases approximately linearly toward the VBM, e.g., as for CdS,^47^ Cu­(In,Ga)­(S,Se)_2_,^48^ or GaAs,^49^ linear extrapolations of the leading edge will yield reliable values for the VBM.^35,38,50^ We therefore stress that, before applying linear extrapolations to determine the VBM (or conduction band minimum), the presence or absence of sharp steps or narrow peaks in the PDOS close to the band extrema needs to be checked.

## 4 Conclusions

In summary, we have investigated the valence electronic and defect structure at three different types of Ga_2_O_3_ surfaces. We have employed PES and HAXPES of a β-Ga_2_O_3_ single crystal, a β-Ga_2_O_3_ single crystal after an Ar^+^-ion treatment, and a Ga_2_O_3_/CIGSe photovoltaic thin-film sample. The measurements were complemented by DFT spectra calculations. To gain depth-resolved information, measurements were performed for excitation photon energies ranging from 0.1 to 6.3 keV. The valence-band spectra of the cleaved β-Ga_2_O_3_ single crystal sample show a pronounced excitation energy dependence that can be well reproduced with the DFT calculations when taking the excitation-energy dependence of the photoionization cross sections into account. Both, the Ar^+^-ion treated β-Ga_2_O_3_ single crystal, as well as the Ga_2_O_3_/CIGSe sample, show significantly broadened valence-band spectra compared to those of the cleaved β-Ga_2_O_3_ single crystal. We ascribe this to a less well-defined long-range order and defect-rich structure at the surface of these samples. In addition, these samples show intensity above the VBM (i.e., in the band gap), which we attribute to acceptor-like defect states. This intensity in the band gap near the VBM region increases for the more surface-sensitive measurements, showing that these states are mainly localized at the surface. We also highlight the importance of DFT-based spectra calculations for determining valence band maxima, especially for materials with a PDOS showing intense and sharp features close to the VBM. The here-presented depth-resolved analysis demonstrates that (and how) PES and HAXPES provide direct insights into the impact of defect states on the electronic structure, which, in turn, can give important input for optimizing charge-carrier transport across interfaces in devices with transparent conductive oxides.

## Data Availability

The data that support the findings of this study are available from the corresponding authors upon reasonable request.

## #

The Institute for Solid State Physics, The University of Tokyo, 5-1-5 Kashiwanoha, Kashiwa, Chiba 277-8581, Japan
